# Ovarian cancer G protein coupled receptor 1 suppresses cell migration of MCF7 breast cancer cells via a Gα_12/13_-Rho-Rac1 pathway

**DOI:** 10.1186/1750-2187-8-6

**Published:** 2013-05-10

**Authors:** Jing Li, Bin Guo, Jing Wang, Xiaoyan Cheng, Yan Xu, Jianli Sang

**Affiliations:** 1 Key Laboratory for Cell Proliferation and Regulation Biology of Ministry of Education, Institute of Cell Biology, College of Life Science, Beijing Normal University, Beijing, 100875, PR of China; 2 Department of Obstetrics and Gynecology, Indiana University, 975 W. Walnut St. IB355A, Indianapolis, IN 46202, USA

**Keywords:** OGR1, MCF7 cells, Cell migration, G*α*_12/13_, Rho, Rac1

## Abstract

**Background:**

Ovarian cancer G protein coupled receptor 1 (OGR1) mediates inhibitory effects on cell migration in human prostate and ovarian cancer cells. However, the mechanisms and signaling pathways that mediate these inhibitory effects are essentially unknown.

**Methods:**

MCF7 cell line was chosen as a model system to study the mechanisms by which OGR1 regulates cell migration, since it expresses very low levels of endogenous OGR1. Cell migratory activities were assessed using both wound healing and transwell migration assays. The signaling pathways involved were studied using pharmacological inhibitors and genetic forms of the relevant genes, as well as small G protein pull-down activity assays. The expression levels of various signaling molecules were analyzed by Western blot and quantitative PCR analysis.

**Results:**

Over-expression of OGR1 in MCF7 cells substantially enhanced activation of Rho and inhibition of Rac1, resulting in inhibition of cell migration. In addition, expression of the Gα_12/13_ specific regulator of G protein signaling (RGS) domain of p115RhoGEF, but not treatment with pertussis toxin (PTX, a Gα_i_ specific inhibitor), could abrogate OGR1-dependent Rho activation, Rac1 inactivation, and inhibition of migration in MCF7 cells. The bioactive lipids tested had no effect on OGR1 function in cell migration.

**Conclusion:**

Our data suggest, for the first time, that OGR1 inhibits cell migration through a Gα_12/13_ -Rho-Rac1 signaling pathway in MCF7 cells. This pathway was not significantly affected by bioactive lipids and all the assays were conducted at constant pH, suggesting a constitutive activity of OGR1. This is the first clear delineation of an OGR1-mediated cell signaling pathway involved in migration.

## Background

OGR1 and related subfamily members GPR4, G2A, and TDAG8 have been shown to have proton-sensing activities [1-5], although in studies using deficient mice, the pH-dependent effects are rather weak, presumably due to redundancy *in vivo*[6-8]. These receptors have also been shown to be modulated by several lysolipids or to mediate oxidized fatty acid signaling [1,2,9-13]. These lipids include sphingosylphosphorylcholine (SPC), lysophosphatidylcholine (LPC), psychosine, and 9-hydroxyoctadecadienoic acid [9-12]. In addition, a constitutive activity of these receptors has been proposed and supported by showing pH- and lipid-independent effects [13,14].

Most G protein coupled receptors (GPCRs) mediated stimulatory effects on cell proliferation, adhesion, migration, and/or invasion, where the mechanisms have been extensively studied. A few GPCRs, on the other hand, mediated inhibitory effects on cellular activities, including cell proliferation and migration, where the mechanisms are much less understood. In particular, activation of somatostatin receptor 2 has a well-documented inhibitory action on tumor growth [15]. To understand these mechanisms is pivotal in developing novel modalities and therapeutics for human diseases. We and others have shown that OGR1 is likely to be an “inhibitory” GPCR. Over-expression of OGR1 inhibits migration of prostate cancer cells *in vitro* and suppresses tumor metastasis i*n vivo*[13]. Recently, Ren *et al*. showed that OGR1 also mediates inhibitory effects on cell proliferation, adhesion, and migration of ovarian cancer cells [16]. However, the downstream effectors of OGR1 have been only minimally studied.

Rho family small GTPases, primarily Rac, Cdc42, and Rho, are well-known for their regulatory roles in actin reorganization and myosin motor function, and thereby in cell motility and migration [17]. Specifically, Rac activity is increased at the leading edge of a migrating cell [18]. This activity drives the actin polymerization that underlies lamellipodia formation and subsequent forward protrusions [19]. Rac activity also directs the formation of focal complexes [20], which provide the traction force needed to tether the cell to the extracellular matrix (ECM) during the contractile events of migration [21,22]. The involvement of Rho in cell migration is more complex. Rho mediates stress fiber formation and cell adhesion [23]. On the other hand, Rho activity has been correlated with decreased protrusion and migration and its effects have been related to its ability to regulate Rac [23-25]. In addition, activation of Cdc42 triggers formation of filopodia and microspikes [26,27].

The current manuscript is focused on the signaling mechanisms of OGR1 leading to cell migration inhibition in cells, not on the pathophysiological role of OGR1 in breast cancer. MCF7 cells were chosen because they do not exhibit endogenous expression of OGR1, and therefore provide a clean background. We show that forced expression of OGR1 attenuated MCF7 breast cancer cell migration *in vitro.* We also present the first evidence that these effects were mediated by the ability of OGR1 to interact with Gα_12/13_ and modulate the small GTPase Rho, which then suppressed the activation of Rac1 that ultimately inhibited cell migration.

## Results

### OGR1 expression inhibited the migration of breast cancer cells *in vitro*

**Figure 1:**
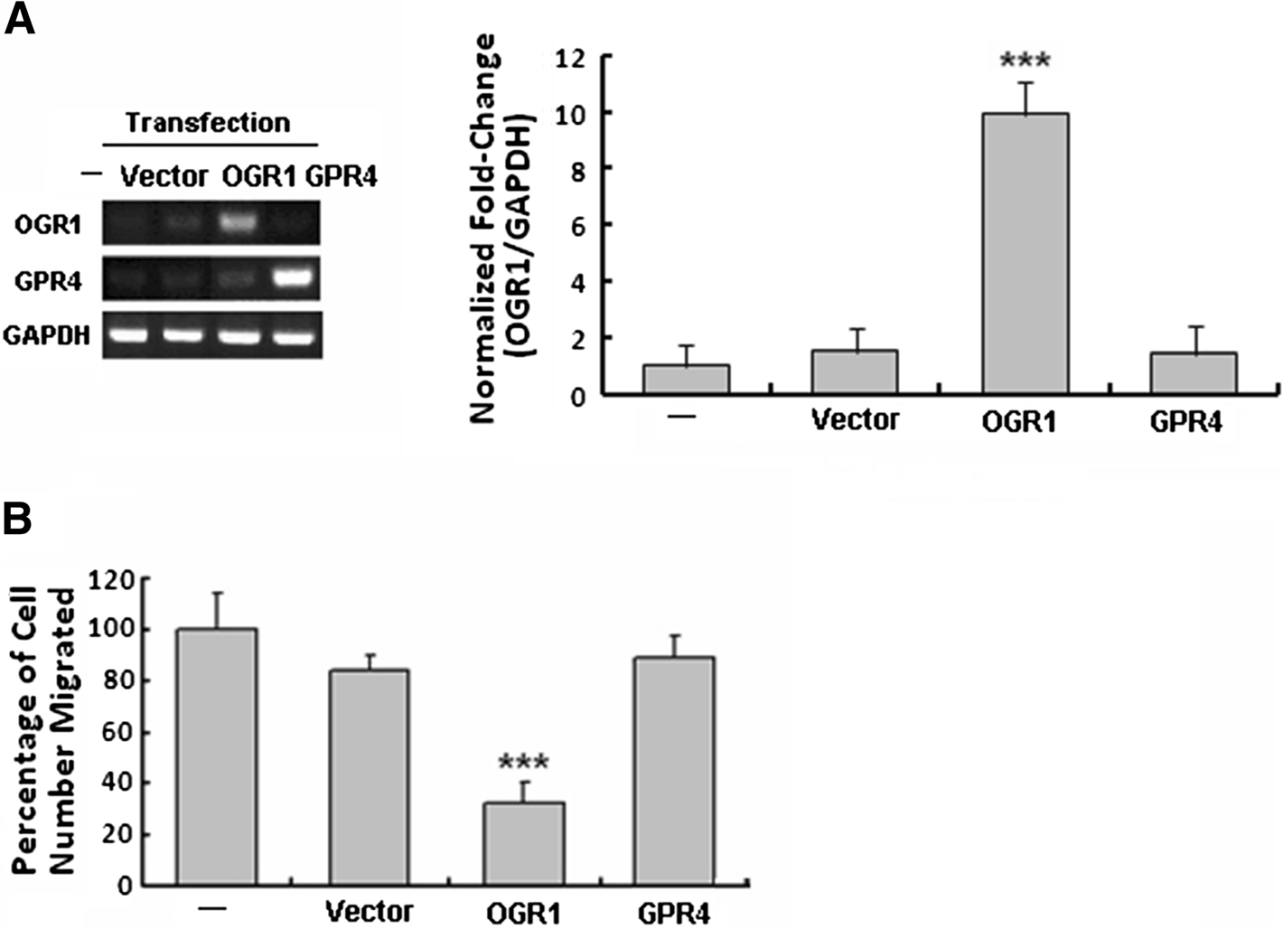
**OGR1, but not GPR4, over-expression inhibited cell migration of MCF7 human breast cancer cells.** Using transiently transfected cells (72 h post-transfection, by RT-PCR and real-time PCR (**A**)), cell migration was analyzed by transwell migration assays (**B**). ***P<0.001. Data are representative of three independent experiments.

When MCF7 human breast cancer cells with very low endogenous mRNA level of OGR1 (Figure 1A) were transfected with the empty vector (control) or vectors containing OGR1 or GPR4 (a GPCR with the highest homology to OGR1), only those cells expressing OGR1 had significantly suppressed migration (Figure 1B), supporting an OGR1-specific inhibitory effect on cell migration.

**Figure 2:**
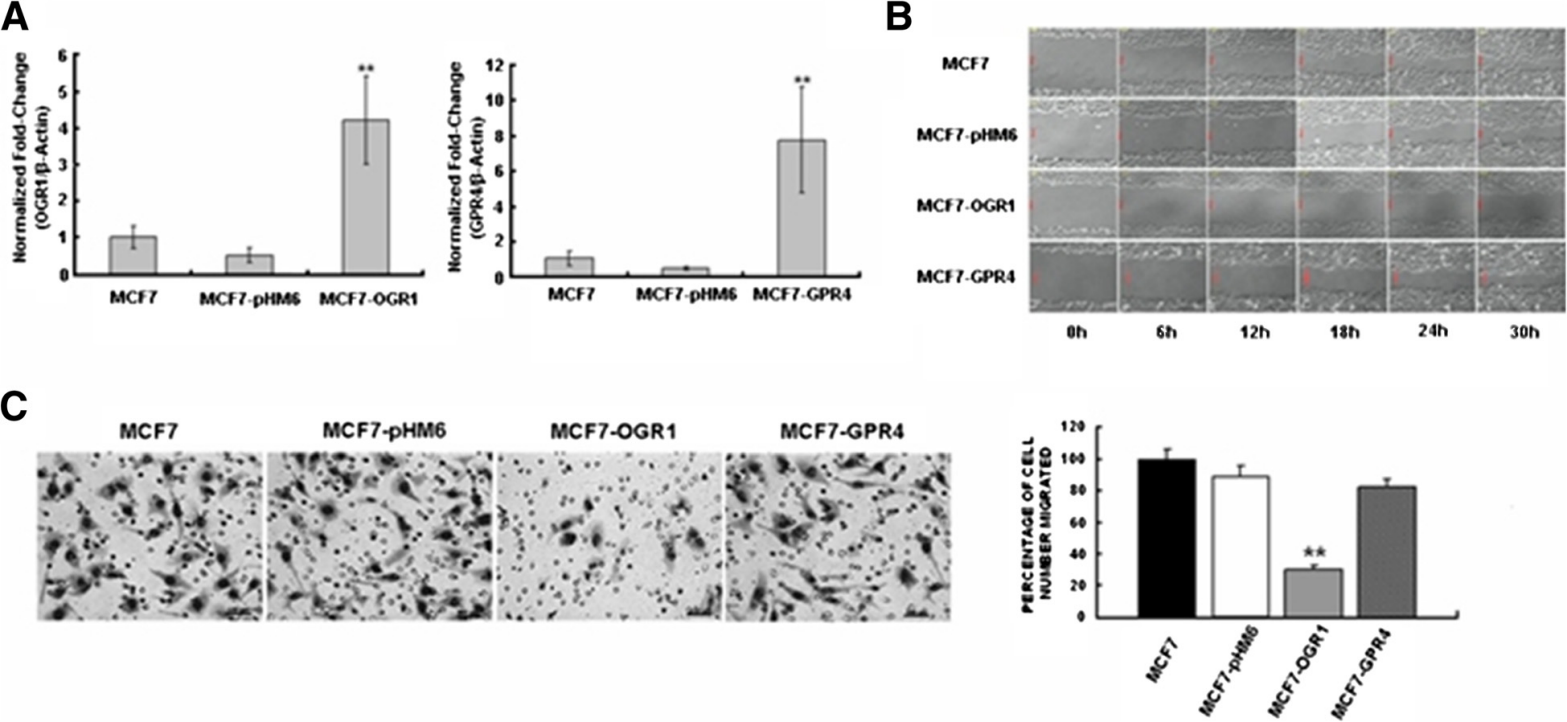
**Stable over-expression of OGR1 inhibited MCF7 cell migration *in vitro*.** (**A**) Identification of MCF7 clones stably over-expressing OGR1 (left panel) or GPR4 (right panel) by real-time RT-PCR. (**B**) The motility of each cell clone was assessed by wound-healing assays. Cells migrated were monitored every hour in a Multi-Dimensional Workstation for Live Cell Imaging (Carl Zeiss). (**C**) Cell migration was analyzed using transwell assays (left). Representative images of cell migrated to the bottom of the inserts of the control cells (MCF7, MCF7-pHM6), MCF7-OGR1, or MCF7-GPR4 cells (left) and the mean percentage of cells migrated (right) are shown. ** *P*<0.01. Data are representative of three independent experiments.

To further study the inhibitory effects of OGR1 on cell migration, stable vector-, HA-OGR1-, and GPR4-expressing MCF7 clones were established. Real-time PCR analyses were performed to identify and confirm these stable clones (Figure 2A). The effects of stably expressed OGR1 and GPR4 on cell migration were assessed by both *in vitro* wound healing assays (Figure 2B) and transwell migration assays (Figure 2C). Consistent with the transient transfection studies, MCF7-OGR1 cells showed significantly reduced migration as compared to the parental, vector-transfected (MCF7-pHM6), or GPR4-transfected (MCF7-GPR4) MCF7 cells (Figure 2B and 2C). Consistent with the results in prostate [13] and ovarian cancer cells [16], GPR4 did not significantly affect MCF7 cell migration even though it shares approximately 54% homology with OGR1 (Figure 2B and 2C). These observations indicate that the cell migration inhibitory effect is specific to OGR1.

### Activation of Rho and inhibition of Rac1 were involved in the inhibitory effect of OGR1 on migration in MCF7 Cells

**Figure 3:**
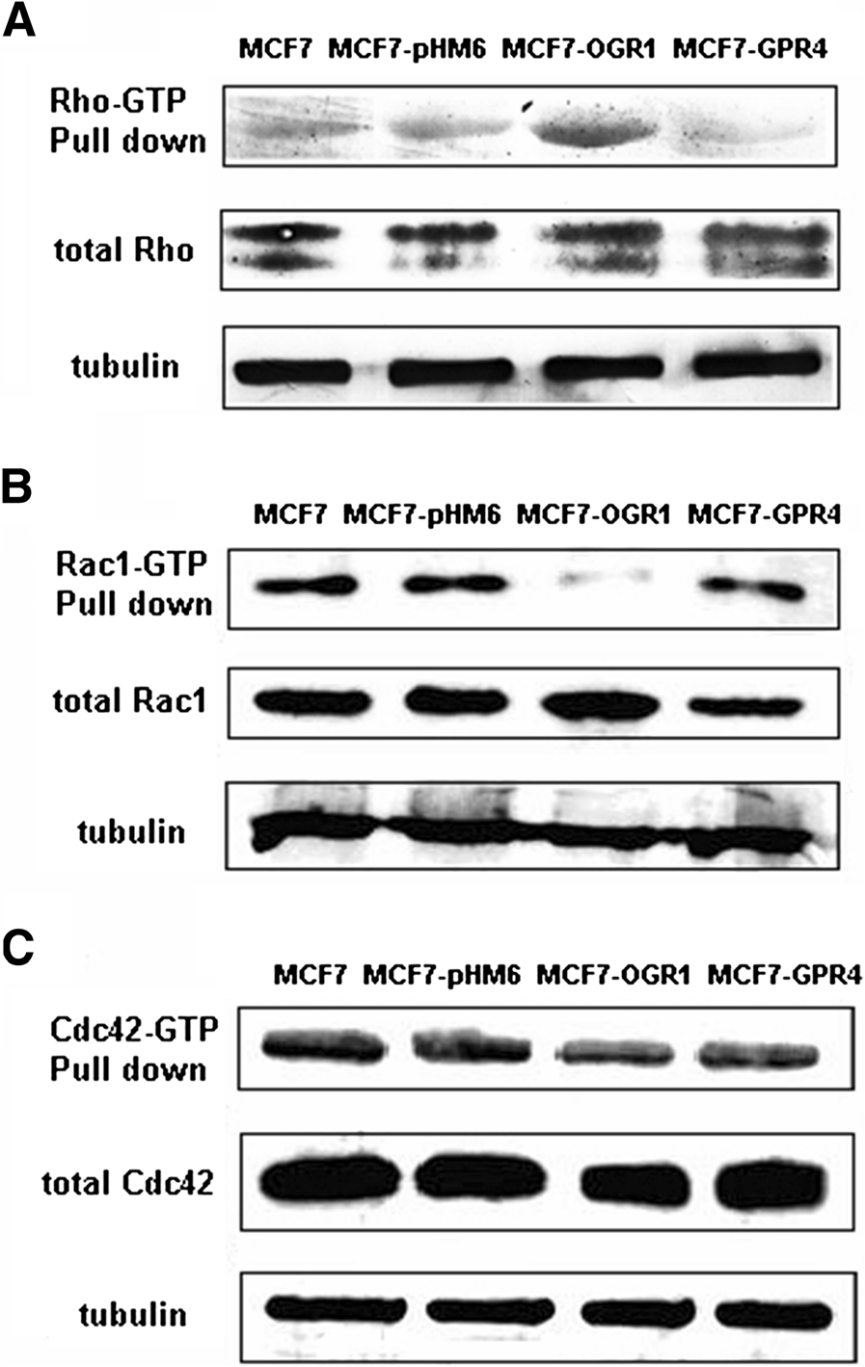
**Effects of OGR1 over-expression on the activity of Rho family members in MCF7 cells.** The activation levels of Rho (**A**), Rac1 (**B**) and Cdc42 (**C**) were examined by pull-down and Western blot analyses as described in Materials and Methods. Total Rho, Rac1, and Cdc42, as well as α-tubulin were analyzed in whole cell lysates. Representative results are from three independent experiments.

To investigate the mechanisms by which OGR1 mediated the inhibition of cell migration, we first tested its effects on Rho, Rac1 and Cdc42 [19-22]. Using Rho-GTP, Rac1-GTP and Cdc42-GTP pull-down assays, we found that the activation of Rho was significantly increased by OGR1 over-expression (Figure 3A). In contrast, Rac1 activity was substantially down-regulated in MCF7-OGR1 cells (Figure 3B). There was no significant change in Cdc42 activation (Figure 3C). Rho and Rac activation were not significantly affected in other control cell lines. These data correlated well with the migratory activities in each cell line (Figure 2).

### OGR1 inhibited breast cancer cell migration in a Gα_12/13_-dependent manner

**Figure 4:**
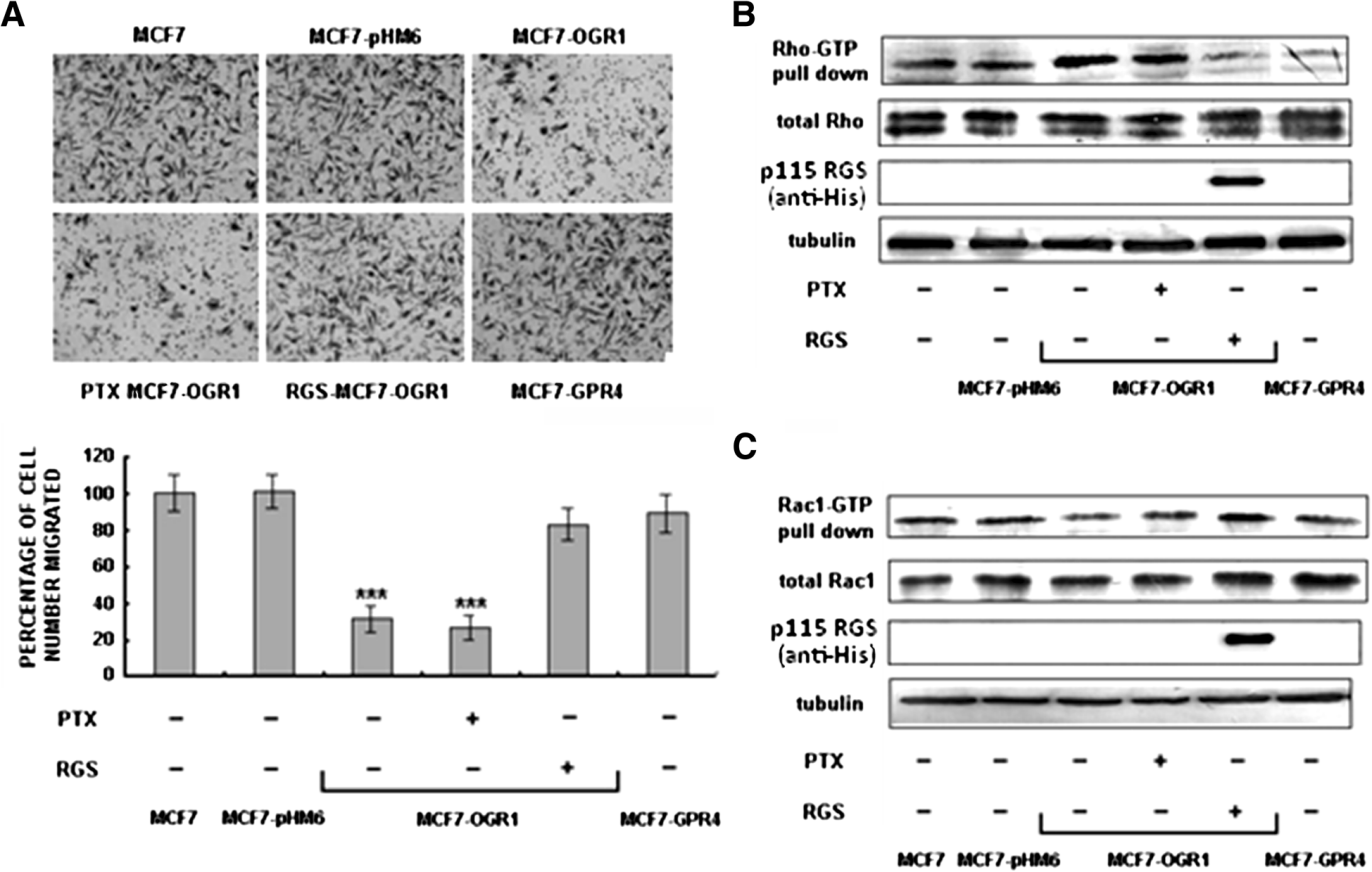
**OGR1 inhibited MCF7 breast cancer cell migration through a Gα_12/13_-Rho-Rac1 pathway.** Cells were pretreated with the solvent or PTX (1 μM) for 16 h, or transfected with the RGS plasmid for 48 h. (**A**) Cell migration was analyzed by transwell migration assays after treatment or using transient transfected cells 72 h post-transfection. ***P<0.001. (**B**) and (**C**) The protein activation levels were examined by pull-down and Western blot analyses as described in Methods. Rho, Rac1, Cdc42 and p115 RGS,as well as α-tubulin were analyzed in whole cell lysates. Representative results are from three independent experiments.

To determine which G proteins might be involved the effects of OGR1, the effects of PTX (a Gα_i_-selective inhibitor) treatment and the transfection of the Gα_12/13_-selective blocker p115RGS on cell migration were tested. Transfection with p115RGS (RGS), but not PTX pretreatment, reversed the inhibitory effects of OGR1 on cell migration (Figure 4A), suggesting that OGR1 acted in a Gα_12/13_-dependent manner.

The levels of activated Rho and Rac1 were analyzed after RGS transfection. RGS over-expression blocked the effect of OGR1 expression on Rho (Figure 4B) and Rac1 (Figure 4C). In contrast, PTX treatment had no effect on Rho or Rac1 activation (Figure 4B and 4C) in MCF7-OGR1 cells. Taken together, these data demonstrate that the Gα_12/13_-Rho-Rac1 pathway is involved in the biological activities of OGR1 resulting in reduced cell migration in MCF7 cells.

### Lysophospholipids (LPLs) did not affect the inhibitory effect of OGR1 on cell migration

**Figure 5:**
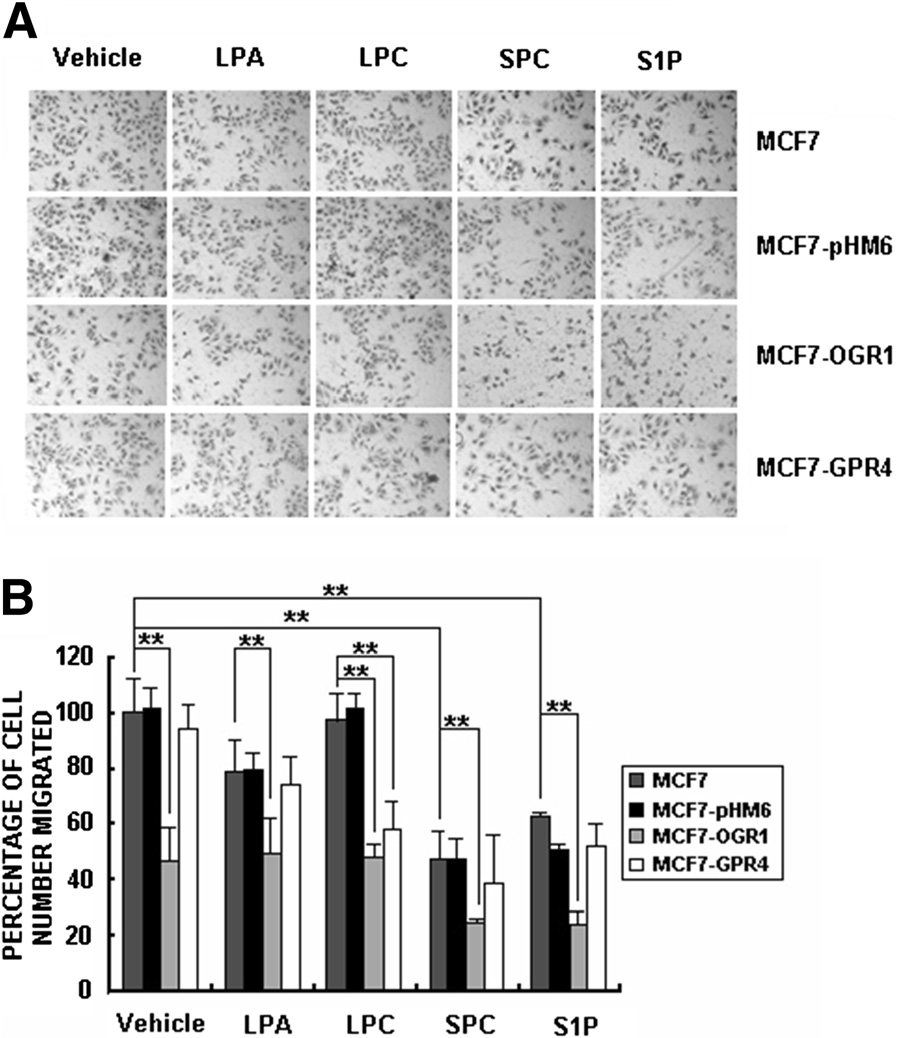
**Lysophoslipids (LPLs) did not modulate the effects of OGR1 on cell migration in MCF7 breast cancer cells.** Cells were treated with vehicle, LPA, LPC, SPC or S1P (all at 2 μM) and cell migration was analyzed by transwell migration assays. Representative images of cell migrated to the bottom of the inserts (**A**) and the mean percentage of cells migrated (**B**) are shown. **P<0.01. Data are representative of three independent experiments.

FBS (10%) was used as a chemoattranctant in all transwell cell migration assays described in this work unless specified. When LPA or S1P (2 μM) was used alone (without FBS) as the chemoattractant, they increased migration of MCF7 cells (5–7 folds) as previously reported [28,29] (data not shown). Since the effects of OGR1 family GPCRs have been shown to be modulated by LPLs [9,10], we tested whether several lysophospholipids, including lysophosphatidic acid (LPA), lysophosphatidylcholine (LPC), sphingosine-1-phosphate (S1P), and sphingosylphosphorylcholine (SPC), could influence the inhibitory effect of OGR1 on cell migration induced by 10% FBS in human breast cancer cells. At 2 μM, SPC and S1P had significant effects on cell migration, but they did not affect the OGR1-induced inhibition of cell migration (Figure 5). LPC had an inhibitory effect on GPR4 over-expressing MCF7 cells (Figure 5), indicating that LPC might inhibit cell migration in a GPR4-dependent manner. However, none of these LPLs modulated the effect of OGR1 on cell migration in MCF7 cells.

## Discussion

OGR1 has been shown to act as a metastasis suppressor gene in a mouse model of prostate cancer [13]. In addition, OGR1 inhibits cell proliferation, adhesion, and migration in ovarian cancer cells [16]. In the present study, we demonstrated that OGR1, but not GPR4, suppressed cell migration of MCF7 cells, extending the migration inhibitory effect of OGR1 to an additional cell lines and cancer type. However, the current study was not focused on the potential pathological role of OGR1 in breast cancer, but rather on the signaling mechanisms by which OGR1 inhibits cell migration. The MCF7 cell line was chosen as a model system since it expresses very low levels of endogenous OGR1. Many classical signaling pathways have been investigated using model systems, for example the commonly used cell lines NIH3T3 and HEK 293. Results obtained from these model systems comprise the core of our knowledge of cell signaling.

Although OGR1 and GPR4 have highly homologous transmembrane domains, their intracellular domains, with which intracellular signaling molecules are expected to interact, are distinct. Wyder L *et al.* have shown that GPR4 is involved in tumor promoting activities [30]. Together with our published studies in prostate cancer cells [13], the results of the current study indicate that OGR1 and GPR4 are likely to have opposing roles in cancer cells, suggesting that they are coupled to different sets of down-stream signaling molecules. The molecular mechanisms underlying this difference remain to be investigated.

The mechanisms by which OGR1 inhibits migration are essentially unknown. In this study, we revealed that the Gα_12/13_ -Rho-Rac1 signaling pathway was activated simply by OGR1 expression. The Rho-Rac family small G proteins play crucial roles in regulating cytoskeleton dynamics and cell migration [17-20]. Rho is required for a migratory response to a variety of growth factors [19,31]. However, under certain conditions, Rho may play a negative role in cell migration. The strong activation of Rho via S1P_2_ receptor-mediated Gα_12/13_ protein, inhibits the migration of CHO cells [32], B16 melanoma cells [33], glioblastoma cells [34,35], mouse embryo fibroblasts [36] and vascular smooth muscle cells [37]. The activation of Rho induced by melatonin [38] and oligodendrocyte lineage transcription factor 2 [39] also inhibits the migration of MCF-7 and U12-1 glioma cells, respectively. We have provided the first evidence showing that OGR1 expression alone increases Rho activation and decreases Rac1 activation. The latter controls membrane ruffling and the formation of lamellipodia, and increases migration [40]. Cdc42 activation was not affected, suggesting that OGR1 may inhibit cell migration by influencing lamellipodia formation. In addition, OGR1-dependent Rho activation and Rac1 inactivation were abolished by the Gα_12/13_-selective blocker p115RGS, supporting an OGR1-Gα_12/13_-Rho-Rac1 signaling pathway. More in-depth signaling studies are needed to further characterize the mechanisms involved in these downstream effects of OGR1.

It has been shown that OGR1 and related GPCRs may have dual functions in mediating signals from either lipids and/or protons [1,2]. SPC, a bioactive lipid molecule, modulates the proton-sensing activity of OGR1. In Chinese hamster ovary cells, SPC inhibits acid-induced activity in a pH-dependent manner [41]. We tested the effects of SPC, as well as other bioactive lysophospholipids, including LPA, LPC and S1P, on the migration of MCF7 cells induced by FBS and found that SPC and S1P had an inhibitory effect on cell migration. Yet, these inhibitory effects appeared to be independent of OGR1 expression and therefore did not bear on the OGR1 pathway under investigation. In addition, the pH of the media in our experiments was not changed. Therefore, it is unlikely that the proton-sensing activity of OGR1 is involved in its inhibitory effect on cell migration.

## Conclusion

In summary, the data presented in this study demonstrate that the *in vitro* inhibitory effect of OGR1 expression on migration of MCF7 breast cancer cells is constitutively active and is related to a Gα_12/13_ -Rho-Rac1 signaling pathway.

## Methods

### Materials

LA *Taq* DNA polymerase, T4 DNA ligase, and restriction endonucleases *Hin*dIII and *Eco*RI were purchased from TaKaRa (Otsu, Japan). RNase I and ethidium bromide were from Sigma-Aldrich (St. Louis, MO, USA). Trypsinase and Vigofect were from Gibco (Carlsbad, CA, USA) and Vigorous Biotechnology Beijing Co. Ltd (Beijing, China), respectively. Pertussis Toxin (PTX) was purchased from ALEXIS Biochemicals (Beijing, China). Protease inhibitor cocktail tablets were obtained from Roche Applied Science (Rotkreuz, Switzerland).

### Plasmid construction and generation of stable clones

The open reading frames (ORF) of OGR1 and GPR4 were amplified by PCR from cDNAs of MDA-MB-231 human breast cancer cells using primers (sense: 5′- TCGAATTCTCGGCCAACCTGCCCG -3′ and antisense: 5′- TAGAATTCGTGGCGACCGGTGGCTAGG -3′ for OGR1, and sense: 5′- TGAAGCTTCACCATGGGCAACC -3′ and antisense: 5′- CAGAATTCGGGGTCCATTGTG -3′ for GPR4). The amplified ORF was cloned into the mammalian expression vector pHM6 with an N-terminal HA-tag. The resulting expression constructs pHM6-OGR1 and pHM6-GPR4 were verified by DNA sequencing (Invitrogen, Beijing, China). Stable MCF7 cell colonies (monoclonal) were selected with 1000 μg/ml G418. Clones expressing OGR1, GPR4 or vector were designated as MCF7-OGR1, MCF7-GPR4, and MCF7-pHM6.

The RGS (the Gα_12/13_ specific regulator of G protein signaling) domain of p115RhoGEF (p115-RGS, amino acids 1–252) was amplified by PCR from cDNAs of HepG2 human hepatocellular liver carcinoma cells using primers (sense: 5′- CCAAGCTTGCCCAGGGAGATGGAAGACTTCGC -3′ and antisense: 5′- GGAATTCCGGGCAGGCTCGTCCGACCG -3′).

### Cell culture

MCF7 human breast cancer cells were cultured in DMEM (Gibco, Carlsbad, USA) supplemented with 10% FBS. MCF7-pHM6, MCF7-OGR1 and MCF7-GPR4 cell clones were cultured in DMEM supplemented with 10% FBS and 500 ng/μl G418 (Merck, Darmstadt, Germany). Cells were grown in a humidified atmosphere containing 5% CO_2_ and 95% air at 37°C. Fresh medium was always added to the cells the day before an experiment.

### Reverse transcriptase-polymerase chain reaction (RT-PCR) and real-time PCR analysis

Total RNAs were extracted and purified from MCF7 cells using the mRNA isolation system (Novagen, Darmstadt, Germany). cDNA was reversely transcribed from mRNA (1 μg) with oligo dT primers and/or random primers, using the AMV transcriptase RT kit (Takara, Otsu, Japan). The synthesized cDNAs (2 μl/reaction) were used as templates for the PCR reactions. PCR primers used were: human OGR1 (sense: 5′-TTCCTGCCCTACCACGTGTTGC-3′ and antisense: 5′-TGGCGAGTTAGGGGTCTGGAAG-3′); GPR4 (sense: 5′-TGGGCAACCACACGTGGGAG-3′ and antisense: 5′-TCCAGTTGTCGTGGTGCAGGAAGTA-3′); and human GAPDH (sense: 5′-ACCTCTATCGGGTGTTCGTG-3′ and antisense: 5′-TTCCTCTTGGAGGTGAGTGG-3′). PCR reactions were carried out by initial denaturation, 1 cycle at 94°C for 5 min, followed by 30 cycles of denaturation (94°C for 30 s), annealing (58°C for 30 s), and extension (72°C for 30 s) with 2.5 units of Promega Go Taq polymerase. This was followed by a final extension step of 72°C for 7 min. At the end of the PCR amplification, PCR products were analyzed by 1.5% agarose gel electrophoresis and Gold-view staining.

Real-time PCR analyses were performed using the following primers: human OGR1 sense: 5′-CACCGTGGTCATCTTCCTG-3′ and antisense: 5′-GGAGAAGTGGTAGGCGTTGA-3′, GPR4 (sense: 5′-TGGGCAACCACACGTGGGAG-3′ and antisense: 5′-TCCAGTTGTCGTGGTGCAGGAAGTA-3′) and human beta-actin sense: 5′-GAAGTCTGCCGTTACTGCCCTGTGG-3′ and antisense: 5′-CCCTTGAGGTTGTCCAGGTGAGCCA -3′. The annealing temperature for the real-time PCR was 60°C for 45 cycles. Beta-actin was amplified as an internal reference. All real-time PCR reactions were performed in a 20 μl mixture containing 1 μl cDNA preparation, 1× SYBR Green buffer (PE Applied Biosystems, Foster City, CA, USA), 4 mM MgCl_2_, 0.2 μM of each primers, 0.2 mM dNTPs mix and 0.025 Unit of AmpliTaq Gold® thermostable DNA polymerase (Applied Biosystems, Foster City, CA, USA). Real-time PCR and quantitations were performed using the BioRad iCycler iQ system and software (BioRad, Hercules, CA, USA).

### Wound healing and transwell migration assays

For the wound healing assays, an area was scraped free of cells with a 20 μl pipette tip and cell migration into the wounded area was monitored every hour using a Multi-Dimensional Workstation for Live Cell Imaging (Carl Zeiss, Oberkochen, Germany).

For the *in vitro* transwell migration assay, cells were cultured to 85-95% confluence (cells were not starved before), trypsinized and washed twice with PBS. Cell culture medium with 10% FBS (500 μl) was added to the lower chambers of a 24-well transwell plate (8.0 μm pore size; Corning Inc, Corning, NY). Cells (10^5^ cells in 200 μl serum-free media) were added to each insert and plates were incubated for 16 h at 37°C. Non-migrating cells were removed with a cotton swab. Migrated cells were fixed in methanol for 30 min and stained with crystal violet (1 mg/mL, Fluka Chemical Corp, Milwaukee, WI) for 30 min at room temperature. Excess stain was removed with water, and the chambers were air-dried. Migrated cells were visualized under the microscope and quantified by counting the number of cells in three randomly chosen fields. The final results were presented as relative percentages with the number of cells migrated in the control wells defined as 100%. At least 5 independent experiments were performed, each in triplicate.

### Rho, Rac1, and Cdc42 activation assay

Rho, Rac1, and Cdc42 activation assays were conducted following the manufacturer’s protocol (Millipore, Billerica, MA, USA). In brief, cells were washed with ice-cold PBS and lysed in a lysis buffer (50 mM Tris–HCl [pH 7.2], 500 mM NaCl, 10 mM MgCl_2_, 1% Triton X-100, 0.5% sodium deoxycholate, 0.1% sodium dodecyl sulfate, 5 μg/mL of leupeptin and aprotinin, 0.1 mM PMSF). Cell lysates were clarified by centrifugation at 13,200 rpm at 4°C for 30 min, and equal volumes of lysates were incubated with GST-p21-activated kinase (PAK) (for determination of Rac and Cdc42 activities) bound to glutathio ne-Sepharose 4B beads (Millipore) at 4°C for 60 min. The beads were washed three times with a washing buffer (50 mM Tris–HCl [pH 7.2], 150 mM NaCl, 10 mM MgCl_2_, 1% Triton X-100, 5 μg/ml of leupeptin and aprotinin, 0.1 mM PMSF). Bound Rho, Rac1 or Cdc42 protein was detected by Western blotting using specific monoclonal antibodies (Millipore, Temecula, CA); total Rho, Rac1, Cdc42 and α-tubulin were detected by whole cell lysate Western blotting.

### Western blot analysis

MCF7 cells were washed three times with ice-cold PBS and lysed in a cell lysis buffer (10 mM Tris [pH 7.7], 150 mM NaCl, 7 mM EDTA, 0.5% NP-40, 0.2 mM PMSF and 0.5 μg/ml leupeptin) for 15 min on ice; the lysate was centrifuged at 14,000 g for 30 min at 4°C and the supernatant was collected. The protein concentration was determined using the BCA™ Protein Assay Kit (Pierce, Rockford, IL, USA). The samples were stored at −20°C until subjected to SDS-PAGE (12% polyacrylamide). The proteins were transferred onto PVDF membranes (Schleicher & Schuell, Dassel, Germany). Western blot analysis was performed using specific antibodies (Santa Cruz Biotechnology, Santa Cruz, CA, USA) to the indicated proteins. The secondary antibodies used were alkaline phosphatase-conjugated anti-mouse and anti-rabbit antibodies (Sigma-Aldrich, St. Louis, USA). The proteins were detected by enhanced chemiluminescence (Promega, San Luis Obispo, CA, USA).

## Abbreviations

OGR1: Ovarian cancer G protein coupled receptor 1; RGS: Regulator of G protein signaling; PTX: Pertussis toxin; LPLs: Lysophospholipids; SPC: Sphingosylphosphorylcholine; LPC: Lysophosphatidylcholine; GPCRs: G protein coupled receptors; LPA: Lysophosphatidic acid; S1P: Sphingosine-1-phosphate; GPR4: G protein coupled receptor 4.

## Competing interests

The authors declare that they have no competing interests.

## Authors’ contributions

Jing Li conducted most experiments described in this manuscript. Bing Guo constructed plasmids and generated stable cell clones, in addition to his work in RT-PCR and real-time PCR assays. Jing Wang conducted part of the experimental work and did major editing of the manuscript. Yan Xu and Jianli Sang have designed and directed the studies and have been responsible for the preparation of the manuscript. All authors read and approved the final manuscript.
